# Quantitative Resistance of Potato to *Pectobacterium atrosepticum* and *Phytophthora infestans:* Integrating PAMP-Triggered Response and Pathogen Growth

**DOI:** 10.1371/journal.pone.0023331

**Published:** 2011-08-11

**Authors:** Alexander Kröner, Gaëlle Hamelin, Didier Andrivon, Florence Val

**Affiliations:** INRA (French National Institute for Agricultural Research), Agrocampus Ouest, Université de Rennes1, UMR (Mixed Research Unit) 1099 BiO3P (Biology of Organisms and Populations applied to Plant Protection), Rennes, France; Charité-University Medicine Berlin, Germany

## Abstract

While the mechanisms underlying quantitative resistance of plants to pathogens are still not fully elucidated, the Pathogen-Associated Molecular Patterns (PAMPs)-triggered response model suggests that such resistance depends on a dynamic interplay between the plant and the pathogen. In this model, the pathogens themselves or elicitors they produce would induce general defense pathways, which in turn limit pathogen growth and host colonisation. It therefore suggests that quantitative resistance is directly linked to a common set of general host defense mechanisms, but experimental evidence is still inconclusive. We tested the PAMP-triggered model using two pathogens (*Pectobacterium atrosepticum and Phytophthora infestans*) differing by their infectious processes and five potato cultivars spanning a range of resistance levels to each pathogen. Phenylalanine ammonia-lyase (PAL) activity, used as a defense marker, and accumulation of phenolics were measured in tuber slices challenged with lipopolysaccharides from *P. atrosepticum* or a concentrated culture filtrate from *P. infestans*. PAL activity increased following treatment with the filtrate but not with lipopolysaccharides, and varied among cultivars. It was positively related to tuber resistance to *P. atrosepticum*, but negatively related to tuber resistance to *P. infestans*. It was also positively related to the accumulation of total phenolics. Chlorogenic acid, the main phenolic accumulated, inhibited growth of both pathogens *in vitro*, showing that PAL induction caused active defense against each of them. Tuber slices in which PAL activity had been induced before inoculation showed increased resistance to *P. atrosepticum*, but not to *P. infestans*. Our results show that inducing a general defense mechanism does not necessarily result in quantitative resistance. As such, they invalidate the hypothesis that the PAMP-triggered model alone can explain quantitative resistance. We thus designed a more complex model integrating physiological host response and a key pathogen life history trait, pathogen growth, to explain the differences between the two pathosystems.

## Introduction

Host resistance was, is and will be of major importance to control epidemic plant pathogens. Van der Plank [1], and a number of authors after him [2], [3], have identified two major types of resistance in plant pathosystems, with reference to the development of disease symptoms. While qualitative resistance often based on pathogen recognition through gene-for-gene interactions and leading to programmed cell death, results in a complete exclusion of the pathogen and prevents its spread in host tissue, quantitative resistance is expressed in compatible interactions and is characterised by a reduced rate of pathogen and symptom development. Although quantitative resistance is widespread, there is still a need for more profound insights into its mechanisms, which remain poorly understood [4].

Plant resistance to pathogens is often described to be the outcome of a co-evolutionary dynamic equilibrium between Pathogen-Associated Molecular Patterns (PAMPs) and effector-triggered induction and suppression of plant defense [5]. In *Solanaceae*, the nature of plant defenses to pathogens does not depend on the type of interaction (compatible or incompatible), but these defenses are generally induced earlier and to a greater extent in incompatible than in compatible interactions [6]. This is particularly true for the activation of phenylalanine ammonia-lyase (PAL) during the interaction of potato tubers with compatible or incompatible races of *Phytophthora infestans* [7].

PAL, the key enzyme of the phenylpropanoid pathway, catalyses the transformation of phenylalanine into cinnamic acid. Cinnamic acid is the core molecule for the synthesis of phenolics, which have been shown to be involved in defense reactions, either as physical and chemical barriers or by acting as signal molecules [8].

The hypothesis underlying this work, consistent with the PAMP-triggered response model, is that quantitative resistance to different pathogens is conditioned by quantitative differences in the kinetics or intensity of the same defense mechanisms induced by either the pathogen or by elicitors it produces. If correct, this hypothesis predicts that resistant host genotypes should generate a higher level of general defense mechanisms than more susceptible ones when challenged by pathogen elicitors. We tested this prediction using two pathosystems involving a single host and two pathogens differing in infectious processes, and focusing on one major general defense pathway and its key enzyme. More specifically, our objective was to show whether there is a relation between the level of resistance to a given pathogen, PAL activity induced by elicitors, and subsequent synthesis of phenolics, and whether this relation is the same for different pathogens.

Our strategy was to determine simultaneously the resistance levels of five potato cultivars against *Pectobacterium atrosepticum* and *P. infestans* and the PAL activity induced by elicitors derived from these pathogens. The experiments were carried out on tubers from distinct batches differing in year of production and time of storage. This experimental system exploits i) differences in levels of quantitative resistance among cultivars of potato (*Solanum tuberosum*) against the biotrophic oomycete *P. infestans* [9] and the pectinolytic bacterium *P. atrosepticum* [10], and ii) earlier evidence that quantitative resistance of potato to these pathogens is modulated by changes in phenolic metabolism during the interaction [4], [11], [12], [13], [14], [15], [16], although conclusive direct demonstration of the antimicrobial properties of total or specific phenolics is still lacking. It was designed to overcome two major limitations of earlier reports relative to the hypothesis at hand. First, because most of these studies are limited to one or two potato cultivars and to a single pathogen, they do not allow to unequivocally discriminate the possible contributions of induced defense from that of the genetic background in the expression of resistance, nor to extrapolate the generality of findings to different pathosystems. Using a range of cultivars with different combinations of quantitative resistance to the two pathogens is intended to separate ‘resistance’ from ‘cultivar’ effects. Second, actual resistance levels were usually not measured directly on the plant material experimented, but deduced from previously published information. Since the physiological state of tubers has been shown to affect both resistance levels to *P. atrosepticum* and *P. infestans* [17] and defense related physiological changes [18], published information might thus not reflect exactly the quantitative resistance of the hosts under the conditions of the experiments. We therefore measured defense and resistance on the same plant material, and repeated the experiments on batches of tubers harvested in different years and stored for different durations, to discern ‘resistance’ from ‘physiological status’ effects.

## Results

### Disease severity caused by *Pectobacterium atrosepticum* and *Phytophthora infestans* varies among potato cultivars

To establish a relative ranking of resistance level among potato cultivars, symptom intensity (rot weight for *P. atrosepticum* and discolorations for *P. infestans*) was measured in artificially inoculated tubers of five cultivars, chosen from previously available data as displaying a range of quantitative resistance to both pathogens (Table 1). Three batches of tubers from different harvests and/or storage durations were used for the experiments.

**Table 1:** Published levels of potato tuber resistance to soft rot (*Pectobacterium atrosepticum*) and late blight (*Phytophthora infestans*) assessed in laboratory tests.

Disease	Ackersegen	BF15	Bintje	Kerpondy	Saturna
Tuber blight (1)	High	Very low to low	Low	High to very high	Medium
Soft rot (2)	Low	Medium to low	Medium	High	ND (3)

(1) From the SASA tests in the EuroPotato database (www.europotato.org).

(2) From Pasco et al. [10]

(3) Not determined.

**Figure 1:**
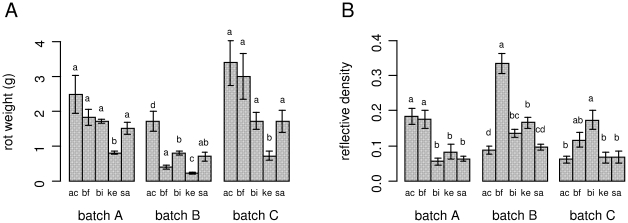
Disease severity caused by *Pectobacterium atrosepticum* and *Phytophthora infestans* in artificially inoculated potato tubers. For *P. atrosepticum *(A), rot weight was measured 5 days after inoculation of half tubers; for *P. infestans* (B), disease severity was expressed as the reflective density deduced with the image analysis software ImageJ from levels of grey recorded on tuber slices scanned 15 days after inoculation with the pathogen. Experiments were performed on tubers from five potato cultivars (ac = Ackersegen, bf = BF15, bi = Bintje, ke = Kerpondy, sa = Saturna), produced in 2008 and 2009. Tubers produced in 2008 were used after 8 months of storage (tuber batch A), tubers produced in 2009 were used after 2 months (tuber batch B) and after 10 months (tuber batch C) of storage. Data for batch C are means from two replicate experiments (vs. one for tuber batches A and B); for each cultivar and treatment, five tubers were used in each experiment. Bars correspond to means ± standard error. Different letters above bars indicate significant differences between means of cultivars within a batch (Tukey's test, p<0.05).

The pathogenicity biotest of *P. atrosepticum* used proved robust, since rot severities did not differ significantly between the two independent experiments (F = 0.0057, P = 0.940) performed on tubers from a single batch (batch C), and no significant ‘experiment x cultivar’ interaction (F = 2.0519, P = 0.089) was detected. There were significantly different levels of soft rot between cultivars (F = 22.5016, P<0.001) and tuber batches (F = 24.1858, P<0.001), but no significant ‘batch x cultivar’ interaction (thus removed from the final model). *P. atrosepticum* caused in all batches the smallest amount of soft rot on Kerpondy, and usually the most on Ackersegen (Figure 1). For each cultivar, rot severity did not differ significantly between the two batches of ‘old’ tubers (batches A and C), and was in most cultivars significantly higher than in the batch of ‘young’ tubers (batch B). Rot severity in ‘old’ tubers of Ackersegen, BF15, Bintje and Saturna did not differ significantly, whereas BF15 had lower levels of soft rot than Ackersegen and Bintje in ‘young’ tubers.

The pathogenicity biotest of *P. infestans* resulted in unstable rankings between cultivars for tuber blight severity among independent replicate inoculations, and generated a statistically significant ‘experiment x cultivar’ interaction (F = 6.6088, P<0.001). This instability was also apparent between different batches, as shown by the statistically significant ‘batch x cultivar’ interaction (F = 11.7613, P<0.001) when all three groups of tubers were considered together. Therefore, the interpretation had to be made separately for each batch.

In all three batches, BF15 had (as expected) the highest blight severity, although it did not differ significantly from all other cultivars in batch C, and from Ackersegen in batch A (Figure 1). The behaviour of Kerpondy and Saturna was also consistent with their expected intermediate levels of susceptibility, although they did not differ significantly from that of Ackersegen in batch C. The main sources for the batch x cultivar interaction stemmed from the erratic behaviour of Ackersegen, and to a lesser extent of Bintje. Ackersegen, deemed to have high tuber resistance to *P. infestans* (Table 1), was unexpectedly susceptible in batch A, but conformed to expectations in batches B and C. In an opposite way, Bintje was generally more resistant than expected, except in batch C.

### PAL activity is induced by a concentrated culture filtrate of *P. infestans*, but not by lipopolysaccharides of *P. atrosepticum*

**Figure 2:**
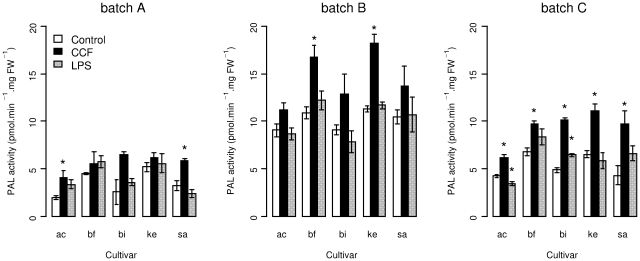
Phenylalanine ammonia-lyase activity in protein extracts from elicitor-treated parenchymatous potato tuber tissue. Activity was measured after 7.5 h of contact with either water as the control (white bars) or elicitors such as a concentrated culture filtrate (CCF) from *Phytophthora infestans* (black bars) or purified lipopolysaccharides (LPS) from *Pectobacterium atrosepticum* (grey bars). Potato cultivars (ac = Ackersegen, bf = BF15, bi = Bintje, ke = Kerpondy, sa = Saturna) used were produced in 2008 and 2009. Tubers produced in 2008 were used after 8 months of storage (batch A), tubers produced in 2009 were used after 2 months (batch B) and after 10 months (batch C) of storage. Data for batch C are means from two replicate experiments (vs. one for batches A and B); in each experiment, for each cultivar and treatment, four disks from different tubers were pooled and aliquoted to constitute four different samples. Bars represent the means ± standard error of phenylalanine ammonia-lyase activity in at least two samples per treatment and cultivar combination. Stars above bars indicate significant differences between the control and tuber tissue treated with the concentrated culture filtrate or lipopolysaccharides, respectively (Dunnett's test, p<0.05).

To induce plant defense, parenchymatous tuber tissue was treated with elicitor preparations: LPS from *P. atrosepticum* and CCF from *P. infestans* (Figure 2).

PAL activity in CCF-treated samples was significantly higher than in LPS-treated samples (t = −4.818, P<0.001) and in water-treated controls (t = 5.817, P<0.001). This induction of PAL by CCF was observed in all cultivars, as shown by the non significant interaction between treatments and cultivars (removed from the final model). By contrast, PAL activity in LPS-treated samples did not differ significantly from that in the water-treated controls (t = 0.999, P = 0.579) (Figure 2). PAL activity was significantly highest in batch B (‘younger’ tubers), lowest in batch A and intermediate in batch C. The relative behaviour of cultivars was generally consistent among the different batches, although a statistically significant interaction was detected in the ANOVA (F = 3.4187, P<0.001). The main source of this interaction was probably due to Bintje, which showed a lower induction of PAL by CCF and even a decreased PAL activity after LPS treatment in batch B, but not in batches A and C.

### PAL activity varies among potato cultivars

Significant differences between PAL activities were observed among cultivars (F = 88.6205, P<0.001), with generally highest levels in BF15 and Kerpondy and lowest levels in Ackersegen (Figure 2). This was true both in control and CCF-treated samples, as evidenced by the non-significant statistical interaction between cultivars and treatments.

### Relations between PAL activity and disease severity depend on the pathosystem

**Figure 3:**
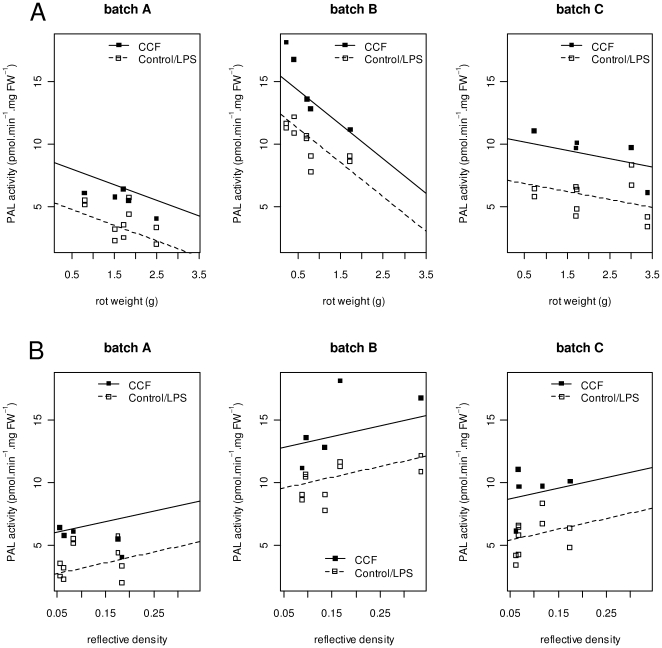
Phenylalanine ammonia-lyase activity in relation to disease symptoms by *Pectobacterium atrosepticum* (A) or *Phytophthora infestans* (B). PAL activity was measured in extracts from tuber tissue treated with water (Control), a concentrated culture filtrate (CCF) from *P. infestans* or purified lipopolysaccharides (LPS) from *P. atrosepticum*. Disease severity caused by *P. atrosepticum* and *P. infestans* was measured in tubers from 5 potato cultivars (Ackersegen, BF15, Bintje, Kerpondy, Saturna). Experiments were performed on 3 tuber batches produced in 2008 and 2009. Tubers produced in 2008 were used after 8 months of storage (batch A), tubers produced in 2009 were used after 2 months (batch B) and after 10 months (batch C) of storage. White boxes represent by-cultivar means of phenylalanine ammonia-lyase activity in replicate samples for Control and ‘LPS treatments, while black boxes represent by-cultivar means of phenylalanine ammonia-lyase activity in CCF treated samples. Results from two experiments on batch C were grouped before calculating means. Lines were drawn using coefficients of linear models fitted to by-cultivar means including results from pathogenicity tests of *P. atrosepticum* and *P. infestans* as co-variable, respectively.

Symptoms of *P. atrosepticum* decreased with increasing PAL activities, whereas symptoms of *P. infestans* increased with PAL activity (Figure 3). The shape of the relationship did not depend on the source of PAL, as shown by identical slopes in CCF-treated and in control (or LPS-treated) samples. The lack of significant interactions in the covariance analysis between treatments and batches (removed from the final model) strongly suggest that these relations are consistent across tubers of different physiological ages. There were no significant differences among the slopes of the regression lines in different batches in *P. infestans*, while the slope of the relationship was significantly higher in the younger tubers (batch B) than in some of the older ones (batch C) for *P. atrosepticum*.

### Disease severity due to *P. atrosepticum*, but not *P. infestans*, is reduced in tubers with pre-induced PAL activity

**Figure 4:**
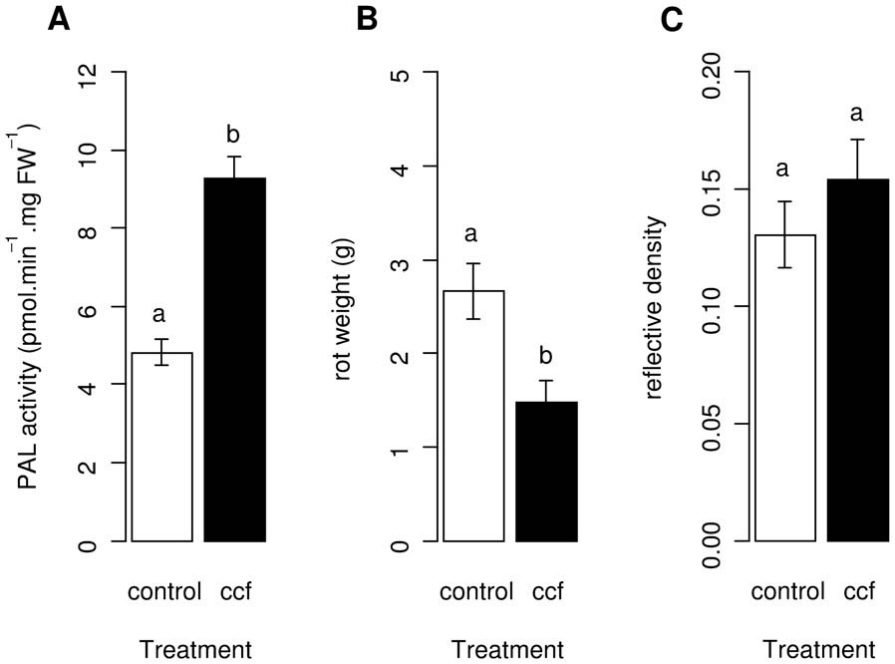
Phenylalanine ammonia-lyase activity and disease severity in potato tubers pre-treated with CCF. Tuber slices of cultivar Bintje were surface-treated with 100 µl of a concentrated culture filtrate (CCF) of *Phytophthora infestans* at a concentration of 400 µg.ml^−1^ or with water as the control. Slices were incubated during 7.5 h at 20°C and subsequently assayed for **Phenylalanine ammonia-lyase** (PAL) activity (A) or inoculated with pathogens. For *Pectobacterium atrosepticum* (B), weight of soft rot developed during 5 days at 20°C was measured. For *P. infestans* (C), disease developed during 5 days at 17°C and is expressed as reflective density. PAL activity was determined in four aliquot samples prepared form pooled tissue of 5 tuber slices and disease severity was determined in 10 tuber slices. Bars correspond to means ± standard error of data from two replicate experiments. Different letters above bars indicate significant differences between means of treatments (Tukey's test, p<0.05).

Pathogenicity tests were carried out on tuber slices from cultivar Bintje where PAL activity was induced by CCF 7.5 h before inoculation. As expected, PAL activity was increased in CCF-treated slices compared to the water treated control (t = 3.476, P<0.01) (Figure 4, A). Increased PAL activity at the time of pathogen inoculation was associated with decreased disease severity caused by *P. atrosepticum* (t = −3.223, P<0.01), (Figure 4, B). However, CCF-treatment did not affect significantly disease caused by *P. infestans* (t = 0.942, P>0.1), (Figure 4, C).

### PAL activity is positively related to subsequent accumulation of phenolics

**Figure 5:**
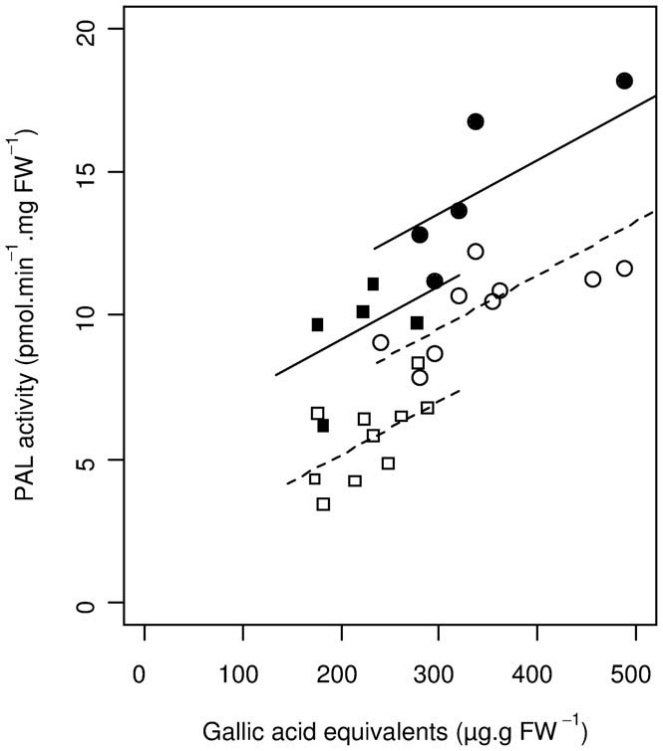
Relation between Phenylalanine ammonia-lyase activity and total phenolic content. Phenylalanine ammonia-lyase (PAL) activity and total phenolic content (expressed as gallic acid equivalents) were measured in extracts from potato tuber tissue treated with water (Control), a concentrated culture filtrate (CCF) from *Phytophthora infestans* or purified lipopolysaccharides (LPS) from *Pectobacterium atrosepticum*. By cultivar means of water and LPS treated samples are represented as white symbols, CCF treated samples as black symbols. Experiments were performed on 5 potato cultivars (Ackersegen, BF15, Bintje, Kerpondy, Saturna) from tuber batches produced in 2009 and used for experiments after 2 months of storage (circles) and after 10 months of storage (boxes). Lines were drawn by using coefficients of a linear model fitted to by-cultivar means for PAL activity and total phenolic content.

Analysis of covariance revealed a significant, positive relation between PAL activity and total phenolic content. This relation was consistent, as shown by identical slopes in all batches and treatments, despite the higher PAL activity in i) ‘young’ tubers from batch B than in ‘old’ tubers from batch C and ii) in the presence of CCF compared to other treatments (Figure 5).

### Chlorogenic acid inhibits in vitro growth of *P. atrosepticum* and *P. infestans*

**Figure 6:**
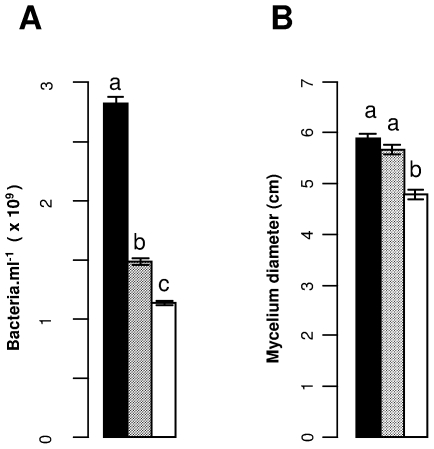
Effect of chlorogenic acid on pathogen growth. Pathogen growth was determined in culture medium enriched with chlorogenic acid to 0, 200 and 350 µg.ml^−1^ . Concentrations of *Pectobacterium atrosepticum* in liquid culture were determined after 40 h of contact (A). Radial mycelium growth of *Phytophthora infestans* from mycelia disks on solid culture medium was measured after 7 days (B). Bars represent the means ± standard error of results from two replicate experiments. Different letters above bars indicate significant differences between treatments (Tukey's test, p<0.05).

Chlorogenic acid, the main phenolic molecule accumulated following CCF treatment (HPLC analysis; data not shown), slowed down the growth of *P. atrosepticum* and *P. infestans* when added to the culture medium at an initial concentration of 350 µg.ml^−1^ (Figure 6). At the lower concentration of 200 µg.ml^−1^, growth inhibition was diminished and only significantly different from the water treated control for *P. atrosepticum*.

## Discussion

The physiological understanding of quantitative resistance of plants to pathogens is still incomplete. Here, we tested the hypothesis that differential induction by pathogen-derived elicitors of a general defense pathway among cultivars is sufficient to explain quantitative host resistance. According to this hypothesis, we expected the pathogen elicitors to induce more PAL activity in the more resistant cultivars, thus leading to reduced disease severity.

**Figure 7:**
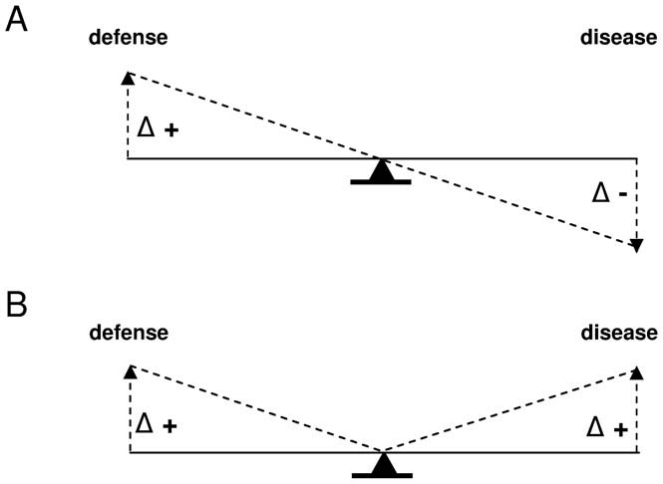
Different defense-disease relations illustrated by a ‘see-saw but see-see’ model. For *Pectobacterium atrosepticum* (A), the model describes the interaction according to the ‘see-saw principle’: increased defense is associated with decreased disease. For *Phytophthora infestans* (B), the model describes the interaction according to the ‘see-see principle’: increased defense is associated with increased disease. A plausible explanation for these differences is proposed by a general interaction model (Figure 8).

We established consistent statistical relations between the level of defense reaction, assessed as elicitor-triggered PAL activity, and quantitative resistance, assessed as the severity of disease in controlled inoculation experiments. However, the type of relation was different in each pathosystem (Figure 7). These relations proved robust in our experimental conditions, as they held for tubers produced in different years and stored for various durations.

While the higher level of defense in the cultivars more resistant to *P. atrosepticum* is consistent with earlier evidence [14], [15], [16] and with our working hypothesis, a higher level of defense was found in the cultivars more susceptible to *P. infestans*. This was surprising given earlier results suggesting phenolic metabolism of potato to be implicated in resistance to *P. infestans* [4], [11], [12], [13], and our own data showing the deleterious effect of chlorogenic acid on pathogen growth.

**Figure 8:**
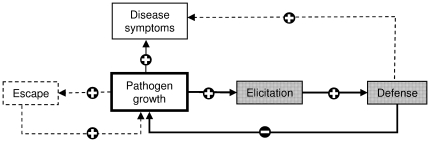
A general interaction model coupling physiological response with key life history traits of the pathogen. The model revolves around a key life history trait, pathogen growth, which drives both physiological (light shaded boxes and bold solid lines) and demographic responses (white boxes and dotted lines). The physiological part of the model derives from the PAMP-triggered response model [5]. It is assumed to occur in all pathosystems; however, defense reactions can also lead to enhance necrosis, and hence to enhanced symptoms of disease – as is the case in *Phytophthora infestans* (dotted lines). Pathogen growth varies between pathosystems, according to host exploitation strategies. In all cases, enhanced pathogen growth leads to more disease; however, in biotrophic pathogens like *P. infestans*, which reproduce on living tissue, growth can lead the pathogen to escape tissue before effective defense reactions take place. This model explains why although the PAMP-triggered response model probably works in all cases, opposite relationships can be observed when plotting the intensity of defense reactions against host resistance (i.e. disease symptoms).

The data for *P. infestans* therefore do not support the idea that the PAMP-triggered active defense is sufficient to explain quantitative resistance – although it does in *P. atrosepticum*. Accounting for the differences between the two pathosystems thus requires a more complex interaction model, coupling physiological response with key life history traits of the pathogen (Figure 8). The physiological component of this model is closely related to the PAMP-triggered immunity model [5], and assumes i) that pathogen growth enhances elicitor production, ii) that elicitors trigger defense reactions – in particular the synthesis molecules with antimicrobial effect. This occurs in both pathosystems studied here. Our model is thus based on one key life history trait, pathogen growth, which drives both the development of disease symptoms and induced defense reactions. However, pathogen growth can also result in pathogen escape from host tissue where defense is triggered. This has been shown for *P. infestans* [4], [19], which produces extending lesions by growing on living host tissue, but to our knowledge not for *P. atrosepticum*, which macerates host tissue and feeds from degraded cells. This major difference in infectious processes could explain why our original hypothesis, restricted to the physiological part of the model, was verified for *P. atrosepticum* but not for *P. infestans*.

Several other results provide additional support for the potential role of pathogen escape in resistance. Firstly, we demonstrated that a physiologically relevant concentration of chlorogenic acid, the major phenolic compound in potato [20], inhibits the growth of both pathogens, *P. atrosepticum* and *P. infestans, in-vitro*. This implies that chlorogenic acid would be effective against both pathogens if there were no possible pathogen escape *in planta*. Secondly, Friend et al. [21] showed that PAL activity was always induced to a greater extent in infected potato tissue than either at the colonization front or in proximal (still non-infected) tissue. This suggests that the growing front of the pathogen occurs in tissue where defense reaction is not yet activated, and that defense occurs after the pathogen has grown further away.

It is interesting to notice that the importance of pathogen escape in quantitative resistance (or susceptibility) to *P. infestans* does not disprove the validity of the physiological component of the model in this case, and hence its general validity. It only implies that the induction of active defense according to the PAMP-triggered immunity model cannot explain alone quantitative resistance in all pathosystems, because it overlooks the existence of additional loops liable to modulate pathogen (and symptom) development.

Further to the ‘escape’ mechanism, other idiosyncrasies of the late blight pathosystem probably also contribute to the positive relationship between PAL activity and disease symptoms. One of these is linked to the fact that CCF components are able to induce necroses, *i.e.* symptoms of disease. Elicitor activity and tissue necrosis are due to the same fraction of the culture filtrate [22], [23], and necrosis due to the filtrate are more pronounced in a cultivar highly susceptible to *P. infestans* than in tubers from a cultivar partially resistant [24]. Furthermore, Bariya et al. [25] found that a culture filtrate of *P. infestans* elicited PAL activity in leaves of susceptible and R-gene resistant potato cultivars. These findings firmly suggest that molecules present in the culture filtrate - and thus secreted by *P. infestans* during colonisation - contribute to the development of disease symptoms as well as to the elicitation of defense reactions, in particular PAL activity.

Two more confusing factors come in play in the model proposed here. First, the pathogen is known to produce much less elicitors *in planta* than in axenic culture [26]. This can most likely lead to a lesser defense activity in the plant, and thus favor pathogen growth. Second, the pathogen may not be sensitive to the secondary metabolites derived from the phenylpropanoid pathways. Our data however show that this is probably not the case in *P. infestans*, which growth is inhibited *in vitro* by chlorogenic acid.

Interestingly, PAL activity was not triggered by LPS alone, although it has been shown to be increased during the interaction with living *P. atrosepticum* cells [27]. Three hypotheses can account for this result: i) LPS is not recognised by the plant cell; ii) LPS is recognised but the signal is not transduced to activate PAL or iii) the bacterium has developed mechanisms in order to minimize recognition of LPS in order to avoid additional defense. The first hypothesis is disproved by earlier reports that LPS triggers early defense pathways (acidification of extracellular medium) in potato cell cultures [28] and by recent experiments that have shown LPS to bind to the extracellular cell wall (F. Val et al., unpublished data). Therefore, it is most likely that recognition of LPS does not lead to PAL induction, either because of a constitutive or induced alteration of signal transduction. This is consistent with inhibition of MAMP-induced signalling by extracellular polysaccharides [29].

Our results highlight the complexity of the interplay between quantitative resistance and defense. They strongly suggest that quantitative resistance depends both from physiological responses well described in the PAMP-triggered response model and to life history traits of the pathogen, leading to pathogen escape from host tissue where defense is active. We think that the integrative approach, combining physiopathology and ecology, that is underpinning the explanatory model proposed here, should prove very useful for the understanding of quantitative resistance, and open new ground for breeding strategies targeting both modes of reducing disease symptoms.

## Materials and Methods

### Plant material

Tubers of five potato cultivars (Ackersegen, BF15, Bintje, Kerpondy, Saturna) with reported differences in resistance levels to *P. atrosepticum* and to *P. infestans* (Table 1) were obtained from the INRA Potato Research Station in Ploudaniel (France).

Tubers from three separate batches differing in production year and storage duration were used for experiments. Batch A contained tubers produced in 2008 and stored at 2°C for 8 months. Batches B and C consisted of tubers produced in 2009 and stored at 2°C for 2 and 10 months, respectively. Therefore, batches A and C are representative of tubers physiologically older than batch B. The timing of experiments imposed to use another batch of tubers of cultivar Bintje, produced in 2010 and stored for 8 months, for the pathogenicity tests on tuber slices in which defense was induced ahead of inoculation.

Prior to use, all tubers were washed in tap water, surface-disinfected by dipping in 70% ethanol, air-dried and subsequently acclimated at ambient temperature for 24 h.

### Inoculum

Inoculum of *P. atrosepticum* (CFPB 5889, INRA Angers, France) was prepared as described by Desender et al. [28]. Bacterial concentrations were adjusted with distilled water to 5×10^8^ cfu.ml^−1^. Inoculum of *P. infestans* (Isolate 08-P15-12, INRA Rennes, France) was obtained as described by Montarry et al. [30] and adjusted to a final concentration of 5×10^4^ sporangia.ml^−1^ before liberation of zoospores.

### Pathogenicity biotests

A relative ranking of tuber resistance to *P. atrosepticum* and *P. infestans* was established by quantification of symptoms after artificial inoculations in controlled conditions, as described by Pasco et al. [10] and Niemira et al. [31]. For *P. atrosepticum*, five tubers of each cultivar were halved longitudinally and 50 µl of inoculum was pipetted in a small well cut with a cork borer into the center of each half tuber. Two control tubers received 50 µl of water instead of inoculum. After 5 days incubation at 20°C in a dark and water-saturated ambience, rotted tissue was collected and weighed. For *P. infestans*, five whole tubers of each cultivar were sprayed with 1 ml of inoculum. A negative control was established with five tubers sprayed with 1 ml of water. After incubation for 15 days at 17°C in a dark and water-saturated ambience, tubers were sliced transversely and the reflective density was measured individually on four sections using a desk scanner (Epson Perfection 1260) calibrated to density with a reflective density tablet (STOUFFER Graphic Art R2110C). Image analysis was performed with the ImageJ software [32]. The reflective density of cut surfaces from control tubers was subtracted from reflective density of cut surfaces from corresponding tubers inoculated with *P. infestans*. Negative values resulting from subtraction were excluded from further analyses.

### Elicitation

A concentrated culture filtrate from *P. infestans* and purified lipopolysaccharides were obtained as described by Desender et al. [28] and adjusted to a final concentration of 200 µg.ml^−1^ for use as elicitors. Slices (20 mm in diameter, 2 mm thick) were prepared from parenchymatous tuber tissue. Slices were cut in half, washed in distilled water, transferred to plastic trays containing moistened filter paper, and kept at room temperature in the dark for 24 h before application of elicitors. Elicitor suspensions (25 µl) were pipetted onto half-slices and spread on the whole surfaces; distilled water was applied to control half slices.

PAL activity was tested in samples of four half-slices per treatment (cultivar x elicitor combination) taken after 7.5 h of contact and ground to a fine powder in liquid nitrogen. The aliquot parts of 300 mg were prepared and stored at −20°C until biochemical analyses were performed.

### PAL activity determination

Proteins from 300 mg aliquots were extracted in 1 ml of 25 mM sodium borate buffer at pH 8.8, supplemented with 10 mg.ml^−1^ polyvinylpyrrolidone and 0.3% (v/v) β-mercaptoethanol. After 15 min of incubation on ice, samples were mixed up in a Retsch Mixer Mill 301, and incubated for further 30 min on ice. Samples were centrifuged for 30 min at 12,000 g. The modified method of Bradford [33] was applied to quantify the proteins in supernatants, using the Bio-Rad Protein Assay Dye Reagent Concentrate (Bio-Rad Cat.-No.: 500-0006). The Bio-Rad standard assay procedure was adopted in order to make up sample volumes of 1 ml.

PAL activity was quantified using a modified method of Zucker [34]. The reaction mixture consisted of 100 µg of protein from samples and 0.017 mmol of phenylalanine, diluted in 1 ml of 25 mM sodium borate buffer at pH 8.8. It was incubated at 40°C for 1 h and absorbance at λ = 290 nm was read against a control without phenylalanine. A molar absorption coefficient of ε = 10 000 L.mol^−1^.cm^−1^ was used to calculate PAL activity, expressed as pmol of t-cinnamic acid produced per min and mg fresh weight (FW). The quantification of PAL activity was performed on two or three aliquots per cultivar x elicitor combination.

### Pathogenicity test in tuber tissue with induced PAL activity

Potato tubers (cultivar Bintje) were cut transversely to obtain 2 mm and 10 mm thick slices. Slices were transferred to plastic trays containing moistened filter paper, and stored for 24 h at room temperature in the dark. A volume of 100 µl of CCF at 400 µg.ml^−1^ was subsequently pipetted onto each slice and spread on the whole surfaces; distilled water was applied to control slices. After 7.5 h at room temperature in the dark, the 2 mm-thick slices were sampled and PAL activity was determined as described above. *P. atrosepticum* and *P. infestans* were inoculated on the 10 mm-thick tuber slices and were placed at 20°C and 17°C, respectively. Disease severity was assessed after 5 days of incubation, as described above, except that for *P. infestans*, where the upper surface of the slice was scanned after having removed a 1 mm thick slice.

### Determination of total phenolics contents

Total phenolics were extracted from 300 mg of grinded potato tuber tissue in 1 ml of methanol containing 1% (v/v) acetic acid. The reaction mixture was incubated at 80°C minutes for three successive steps of 5 min, separated by grinding in a Retsch Mixer Mill 301. The supernatant containing soluble phenolics was separated from debris by centrifugation at 5,000 g during 10 minutes. The extraction process was repeated and supernatants were merged. Total phenolic compounds were quantified using the method initially proposed by Folin and Ciocalteu [35] with several modifications. The reaction mixture was prepared by successively adding to a haemolysis tube containing 2 ml of distilled water 0.5 ml of each of the following components: phenolic supernatants, Folin-Ciocalteu's Phenol Reagent 2 N (Sigma-Aldrich, F9252), aqueous solution of sodium carbonate (c = 200 µg.ml^−1^) and distilled water. It was incubated at ambient temperature for 90 minutes and absorbance was determined spectrophotometrically at λ = 760 nm. The total content of soluble phenolics was calculated by comparison with a standard curve of gallic acid, and is expressed as µg of gallic acid equivalents per g FW.

### Antimicrobial test of chlorogenic acid

Chlorogenic acid was added to culture media (King B or pea agar), to reach final concentrations of 200 and 350 µg.ml^−1^. Culture medium without chlorogenic acid served as the control. For *P. atrosepticum*, amended liquid King B medium containing 6×10^8^ bact.ml^−1^ was placed on a rotary shaker at 300 rpm. After 0, 20 and 40 h at room temperature, bacterial density was assessed spectrophotometrically as described by Desender et al. [28]. For *P. infestans*, melted pea medium containing 1.5% of agar was chilled down to 40°C, enriched with adequate amounts of chlorogenic acid and transferred to Petri dishes. Mycelial disks were placed in the centre of each Petri dish and incubated at 17°C. After 7 days, radial growth of mycelium was measured in orthogonal directions. Measurements were repeated on 5 Petri dishes for each experimental condition.

### Data analysis

All statistical analyses were performed with the statistical software R GUI version 2.10.1 by the R Development Core Team [36]. Null hypotheses were rejected if p<0.05. Data were transformed to natural logarithms or square roots when necessary before performing the analysis of variance and multiple comparisons of means with the Tukey and Dunnett tests. For analysis of co-variance, by-cultivar means were calculated from pathogenicity and biochemical data (enzyme activity or phenolic contents) for each tuber batch. On batches A and B, these are means of disease severity from different tubers or from repeated analysis of PAL or total phenolic content. On batch C, results from two replicate experiments were grouped together before calculating means. Non-significant interactions or factors were removed by backward model simplification, and factor levels not significantly different were grouped together.
